# A colonoscopy quality improvement intervention in an endoscopy unit

**DOI:** 10.1038/s41598-022-04786-y

**Published:** 2022-01-17

**Authors:** Rémi Palmier, Thibault Degand, Serge Aho, Côme Lepage, Olivier Facy, Christophe Michiels, Sylvain Manfredi

**Affiliations:** 1 Département d’hépato Gastroentérologie et Endoscopie Digestive, Centre Hospitalier Universitaire de Dijon, Dijon, France; 2 Département d’hygiène Hospitalière, Centre Hospitalier Universitaire de Dijon, Dijon, France; 3 Département de Chirurgie Digestive et Oncologique, Centre Hospitalier Universitaire de Dijon, Dijon, France; 4 Hepato-Gastroenterology Unit, University Hospital, Dijon, France

**Keywords:** Colonoscopy, Health policy

## Abstract

Many studies identified colonoscopy quality indicators in order to improve performance and safety. We conducted a colonoscopy improvement study. Our study was designed according to a Plan-Do-Study-Act cycle: first recording of our quality indicators and identification of shortcomings, second identification of improvement targets and implementation of new procedures, third second recording of quality indicators, fourth validation of procedures and identification of new goals. Quality indicators derived from European and French guidelines were recorded before and after our improvement actions. We were mainly interested in the quality indicators of the colonic preparation, the description of the diagnosed lesions and on the examination reports. The data of 134 patients prospectively included in January–February 2017 were compared to 133 patients included in May–June 2019, after implementation of improvement procedures, in the digestive endoscopy unit of the university hospital of Dijon, France. Our intervention, and in particular the implementation of new standardized forms, improved preparation quality: Boston Bowel Preparation Scale scores increased significantly from 7.8 to 8.2. Cecal intubation rate increased by 6%, and more adenomas were diagnosed and removed (+3.3%). Adenoma detection rate increased significantly from 26 to 42%. The completion of withdrawal time measure improved from 6.7 to 100%. Our study led to the rapid implementation of corrective actions and improved quality in our unit and in our personal practice. This quality improvement strategy could be easily implemented in every digestive endoscopy unit.

## Introduction

Colonoscopy is the reference examination for the screening, diagnosis, treatment and surveillance of colorectal diseases. Many recent studies have identified several pre- per- and post-procedure factors related to colonoscopy quality, and these factors have been included in French (French Society of Digestive Endoscopy: SFED) and European guidelines (European Society of Gastrointestinal Endoscopy: ESGE)^1–5^. These quality indicators aim to improve performance and patients’ safety. In order to improve colonoscopy quality in the Dijon University Hospital digestive endoscopy unit, we carried out a quality improvement study between January 2017 and June 2019, in accordance with the latest ESGE^6^ and SFED^7–9^ quality indicators. We present our intervention according to the SQUIRE guidelines.

## Materials and methods

The study was a quality improvement initiative and was divided into four phases according to a Plan-Do-Study-Act (PDSA) cycle: first, we recorded our baseline quality indicators, identified shortcomings, established improvement targets and created new tools and procedures; second, we implemented these new tools and procedures; third, we recorded the same quality indicators and compared the results with those of the first period; fourth, we validated the improvement measures and identified new goals.

### Baseline record, identification of factors that could be improved and elaboration of new tools and procedures

We included all adult patients referred for a complete screening, diagnostic or therapeutic colonoscopy, with or without general anesthesia at the Dijon University Hospital digestive endoscopy unit between January and February 2017. Patients referred for sigmoidoscopy or for partial colonoscopy were excluded. All colonoscopies were performed by skilled senior practitioners. The factors we considered implementing for our quality improvement project were the quality indicators of the European and French colonoscopy quality guidelines. In accordance with the 2017 ESGE and 2018 SFED guidelines^6–10^, we separated quality indicators into three groups: “pre-procedure”, “per-procedure” and “post-procedure”. We added several quality indicators considered important in our practice: tolerance to the bowel cleansing, evaluated using a questionnaire: Very good/Good/Medium/Bad/Very bad; time to colonoscopy for all patients according to the indication: in particular times between the consultation with the primary care physician, the gastroenterologist, the anesthesiologist, the Fecal immunologic test (FIT) result and the colonoscopy. Time to colonoscopy did not appear as a quality indicator in reference studies, but we considered it a major point, especially for screened patients with a positive fecal immunologic test. The SFED strongly recommends a maximum of 31 days between the FIT gastroenterology visit and the colonoscopy, followed by a printed *day-0 mail*. The quality indicators and objectives are summarized (Appendix):

After analyzing phase 1 results, we identified weaknesses and set improvement targets. We also created new tools and procedures in accordance with national validated recommendations. The main deficiency identified was the quality of the colonic preparation. The interventions implemented were targeted on the harmonization of prescriptions and protocols for colonic preparation, the collection of the quality of the preparation and its evaluation.

### Second phase: implementation of improvement measures

First, we created electronic standardized bowel preparation protocols with simplified order forms available for all practitioners of Dijon University Hospital. These were based on last ESGE, SFED and French society of anesthesiology (SFAR) recommendations^9,11–15^: all common preparation protocols were available, but we encouraged the use of PEG (Polyethylene glycol) 2 L + ascorbic acid. All protocols provided split dosing and if possible, end of take between 3 and 5 h before the colonoscopy.

Second, we created an electronic standardized endoscopic report based on the most recent SFED recommendations^7,8^, which replaced our old free-text examination report. The main indicators we focused on were withdrawal time, BBPS and polyp characterization. The histology order form was automatically generated according to the information provided in the colonoscopy report. Our aim was also to generate an automatic, printed, standardized colonoscopy report with a *day-0 mail* for outpatients. In parallel, practitioners were encouraged to train themselves in the use of quality indicators to improve polyp detection and macroscopic analysis. The new documents and procedures were drawn up and developed with all the unit's endoscopists and implemented at the end of 2018.

### Third phase: second recording of quality indicators

We conducted a final evaluation of our practices in May and June 2019, using the same quality indicators. We compared the results before and after the intervention to identify the impact on the different quality indicators.

### Fourth phase: validation of improvement measures and identification of new goals

#### Statistical methods

Continuous variables were expressed as means with standard deviations except for time variables which were expressed as medians with interquartile ranges (IQR) and categorical variables as percentages. Comparison between baseline and final evaluation were performed using Pearson Chi-square test or Fischer exact tests, when appropriate, for qualitative variables, and using Wilcoxon or Kruskal–Wallis tests for quantitative variables. We collected data prospectively and statistics were validated by the statistics unit of our University Hospital. Statistics were done with STATA® and SAS 9.4® software. p-values and p-trends were considered statistically significant based on the 2-sided probability of 0.05.

### Ethical consideration

In accordance with French law, only oral consent of the participants is necessary and has been obtained.

## Results

### Population characteristics

One hundred and thirty-four patients referred for complete colonoscopy were included in the baseline phase between January and February 2017. One hundred and thirty-three patients were included with the same criteria in the final phase between May and June 2019. Patients’ characteristics are resumed in Table 1. Patients of phase 1 and 2 were comparable for age, sex and body mass index (BMI). Most patients were outpatients 66.4% and 68.5% respectively. Patients’ origins in phase 1 were comparable to those in phase 2.

**Table 1:** Population characteristics.

	Phase 1		Phase 2		*P*
	n = 134	(%)	n = 133	(%)	
Men	73	54.5	66	49.6	0.43
Women	61	45.5	67	50.4	
**Unit**					
Outpatients	89	66.4	91	68.5	0.06
GE inpatients	22	16.3	32	24.0	
Non GE inpatients	23	17.3	10	7.5	
**Applicant practitionner**					
General practitioner	40	29.9	57	42.9	0.06
Own gastroenterologist	53	39.5	37	27.8	
Other gastroenterologist	1	0.7	3	2.2	
Other specialist	40	29.9	36	27.1	
**Colonoscopy indication**					
FIT	30	22.4	6	4.5	0.02
Symptoms	52	38.8	48	36.1	
IBD	15	11.2	17	12.8	
Personal history	11	8.2	26	19.6	
Familial antecedent	15	11.2	13	9.8	
Other (before surgery)	11	8.2	23	17.2	

	Mean	SD	Mean	SD	*p*
Age (years)	59.9	15.4	57.9	14.5	0.17
BMI (kg/m^2^)	26.6	6.2	26.2	6.6	0.8

FIT: Fecal immunological test, IBD: inflammatory bowel disease, BMI: body mass index, SD: standard deviation, GE: gastroenterology.

### Pre-colonoscopy indicators

All patients in the study had a dedicated specialist visit before the colonoscopy and a clear indication was available for all colonoscopies. Colonoscopy indications in phase 1 were significantly different from those in phase 2 with respectively more colonoscopies after a positive FIT: 22% and 4.5%, fewer colonoscopies for personal history: 8% and 20% and fewer colonoscopies for other indications: 8% and 17% (*p* = 0.02). The proportion of colonoscopies for symptoms (anemia, abdominal pain, hemorrhage and diarrhea) was similar in the two phases: 38% and 36% (Table 1). All patients provided consent, and all endoscopy checklists and endoscope disinfections were validated for the two phases.

At the baseline measurement step, a multitude of non-standardized preparation protocols were used by gastroenterologists and no standardized colonoscopy preparation order form was available. We identified this element as an indicator to be improved and we created and implemented an electronic standardized colonoscopy preparation order set with various alternatives, but favoring PEG + sodium picosulfate and split dosing. This standardized electronic order set included diet procedures for the 3 days before the colonoscopy. In phase 1, the most frequent preparations used were PEG + ascorbic acid in 68 (50.7%), PEG only in 36 (26.9%) and sodium picosulfate in 25 patients (18.6%). The results were significantly different after implementation of the standardized bowel preparation protocols with an increase in PEG + ascorbic acid, used in 115 patients (86.5%), and a decrease in both PEG alone, used only in two patients (1.5%), and sodium picosulfate, used in 11 patients (8.3%) (*p* = 0.0001). Split dosing significantly increased from 27 to 97% (*p* = 0.0001), and the last take between 3 to 5 h before the procedure increased from 25 to 95% (*p* = 0.0001). The acceptability level questionnaire was proposed to more than 50%, and almost all of these patients (90%) reported tolerance to the preparation as good or very good in both phases.

The results for bowel preparations and time to colonoscopy or visit intervals are presented in Table 2.

**Table 2:** Bowel preparations and time to colonoscopy.

	Phase 1		Phase 2		*p*
	N = 134	(%)	N = 133	(%)	
**Preparation**					
PEG 4 L	36	26.9	2	1.5	0.0001
PEG 2 L + ascorbic acid	68	50.7	115	86.5	
Sodium phosphate drinkable	3	2.2	0	0.0	
Sodium phosphate tablets	1	0.8	3	2.2	
Sodium Picosulfate	25	18.6	11	8.3	
Enemas	1	0.8	2	1.5	
**Splitting**					
None	24	17.9	4	3	0.0001
2 takes one day before	74	55.2	0	0	
Split dosing	36	26.9	128	97	
**Schedule**					
Morning	122	91	113	85	0.13
Afternoon	12	9	20	15	
**End of take**	n = 105		N = 131		
3–5 h	26	24.8	125	95.4	0.0001
5–8 h	3	2.8	3	2.3	
> 8 h	76	72.4	3	2.3	
**Acceptability**	n = 70		69		
Excellent	3	4.3	8	11.6	0.9
Good	59	85.3	56	81.2	
Middle	5	7.1	3	4.3	
Bad	3	4.3	2	2.9	
Very bad	0	0.0	0	0.0	

Times (days)	N = 132		N = 133		*p*
	Median	(IQR)	Median	(IQR)	
Addressing practitioner—GE	18	[7–31]	36.5	[13–63]	0.0001
GE—anesthesiologist	22	[9–36]	37	[21–49]	0.0003
GE—colonoscopy	38	[2–52]	53	[35–64]	0.0001
**For FIT**	N = 30		N = 6		
FIT—GE	33.5	[27–42]	52.5	[40–81]	0.1
Addressing practitioner—GE	20	[15–28]	35	[28–60]	0.07
GE—Colonoscopy	36.5	[26–39]	52	[32–63]	0.1
FIT—Colonoscopy	69	[58–97]	108	[103–136]	0.02

FIT: Fecal immunological test, GE: gastroenterologist, IQR: interquartile range, PEG: polyethylene glycol.

The median time between the gastroenterologist consultation and the colonoscopy was 38 days (IQR 2-52) in phase 1 versus 53 days (IQR 35-64) in phase 2 (*p* = 0.0001). In the FIT population (n = 30), the median time between the fecal immunologic test result and the colonoscopy was 69 days (IQR 58-97) in phase 1 versus 108 days (median, IQR 103-136) in phase 2, with a significant difference (*p* = 0.02).

### Per-colonoscopy indicators

**Figure 1:**
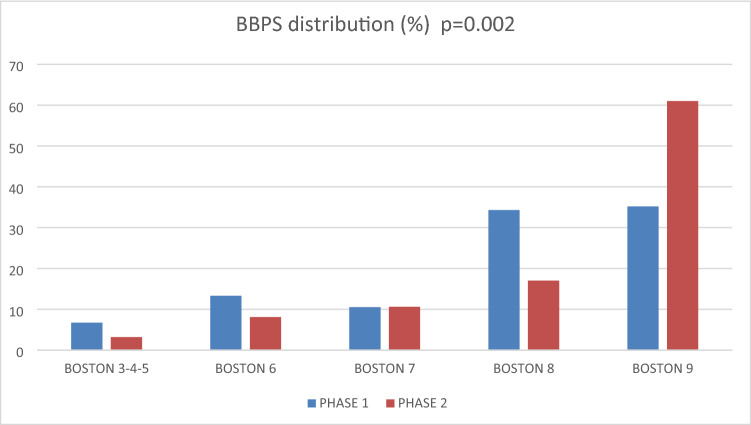
Boston Bowel Preparation Scale distribution.

At the 1st phase, the BBPS was not systematically entered in the colonoscopy report and left free to the choice of the endoscopist. We implemented the requirement to fill in this item in our standardized report, resulting in a BBPS completion increase from 84 to 99% (*p* = 0.0001) (Table 3). BBPS score distribution is shown in Fig. 1. Obstruction was not considered failure and was excluded from the BBPS analysis. There were five obstructions diagnosed in phase 1 (3 cancers and 2 diverticulosis) and three in phase 2 (1 cancer and 2 diverticulosis). BBPS greater than 5 (ESGE cut off) increased from 93 to 97% (*p* = 0.002), and BBPS greater than 6 (SFED cut off) from 80 to 88% (*p* = 0.002). The average BBPS was significantly better in phase 2 (8.2) than in phase 1 (7.8) (*p* = 0.007). There was no difference in BBPS between morning and afternoon colonoscopies with a score of 8.11 versus 7.76, respectively (*p* = 0.8). BBPS was similar whatever the time between the end of the take and the colonoscopy time: less than 5 h or more than 5 h (*p* = 0.23). The best preparation results were obtained with PEG + ascorbic acid, PEG alone and sodium picosulfate with average BBPS at 8.2, 7.4 and 6.9, respectively (*p* = 0.0004). For these three preparations, split dosing was associated with an increase in the BBPS (*p* = 0.03) (Table 3). The cecal intubation rate increased non-significantly from 84 to 90% (*p* = 0.15) (Table 3). Colonoscopy failed in 16 patients in phase 1, four for technical reasons and 12 for insufficient preparation. In phase 2, 10 colonoscopies failed, six for technical reasons and four for insufficient preparation.

**Table 3:** BBPS and colonoscopy results.

	Phase 1		Phase 2		*p*
	n	(%)	n	(%)	
**BBPS**	**125**		**133**		
Completion in report	105	84	123	99	0.0001
**Guidelines objectives**	**105**		**123**		
BBPS > 6 (SFED)	98	93	119	97	0.002
BBPS > 7 (ESGE)	84	80	109	88	
**Average BBPS**	**105**		**123**		
	7.76		8.22		0.007
**Colonoscopy results**	**134**		**133**		
Normal	44	32.8	50	37.6	0.02
Polypes	50	37.3	54	40.6	
Malignancy	7	5.2	1	0.8	
Diverticulosis	15	11.2	11	8.3	
Active IBD	8	6	15	11.3	
Others*	10	7.5	2	1.5	
**Cecal intubation**					
Yes	113	84.3	120	90.2	0.15
No	21	15.7	13	9.8	
Failure	**16**	**12**	**10**	**7.5**	
Technical reasons	4	25	6	60	0.09
Bad preparation	12	75	4	40	

BBPS: boston bowel preparation scale, SFED: French society of digestive endoscopy, ESGE: European society of gastrointestinal endoscopy, IBD: inflammatory bowel disease.

*2 angiodysplasia, 2 infectious colitis, 2 post-radiation colitis, 1 ischemic colitis and 3 unclassified colitis. Significant values are in bold.

Of the 134 colonoscopies performed in phase 1, 44 (32.8%) were normal; polyps were found in 50 cases (37.1%), cancer in seven cases (5.2%), diverticulosis in 15 cases (11.2%), IBD in eight cases (6%) and another diagnosis in 10 cases (7.5%) (2 angiodysplasia, 2 infectious colitis, 2 post-radiation colitis, 1 ischemic colitis and 3 unclassified colitis). Of the 133 colonoscopies performed in phase 2, 50 (37.6%) were normal, 54 (40.6%) revealed polyps, one case of cancer (0.8%), IBD in 15 cases (11.3%), diverticulosis 11 cases (8.3%) and another diagnosis in two cases (1.5%) (Table 3).

The number of colonoscopies done under general anesthesia was similar in both phases: 93% and 98% (*p* = 0.08).

During the 1st phase, the decision to describe the polyps according to the Paris and Kudo classifications was left free to the endoscopist. Following phase 1, we included these items in our electronic report. Completion of the Paris polyp classification was done in only 56.4% of cases in phase 1, increasing to 92.7% of cases in phase 2 (*p* = 0.0001). The Kudo polyp classification was poorly reported in both phases, 9% and 11%, respectively (*p* = 0.3). The polyp retrieval rate was 100%.

Polyp pathology was significantly different in the two phases; there were more hyperplastic polyps in phase 1: 32.7% versus 10.9%, and more adenomas in phase 2: 87.3% versus 54.5% (*p* = 0.003) (Table 4). The polyp detection rate was not significantly different between phase 1 (37%) and phase 2 (41%) (*p* = 0.5). The adenoma detection rate (calculated for the endoscopy unit) was significantly higher in phase 2 (41.7%) than in phase 1 (25.8%) (*p* = 0.01) (Table 4). For FIT indications, the adenoma detection rate was 50% (30 colonoscopies) in phase 1 versus 83% in phase 2 with only six colonoscopies (*p* = 0.12).

**Table 4:** Histology and adenoma detection rate.

	Phase 1		Phase 2		*p*
	n = 131	(%)	n = 132	(%)	
**Histology**	**55**		**55**		0.003
Hyperplastic	18	32.7	6	10.9	
Adenoma	30	54.5	48	87.3	
Adenocarcinoma	7	12.8	1	1.8	
Detection rates*	**116**	**(%)**	**115**	**(%)**	
PDR	55	38.0	55	41.0	0.5
ADR	30	25.8	48	41.7	0.01
FIT detection rates	**30**		**6**		
ADR	15	50.0	5	83.3	0.12

*After exclusion of patients with IBD and emergency situations (n = 15 in phase 1 and n = 17 in phase 2).

PDR: Polyp detection rate, ADR: adenoma detection rate. Significant values are in bold.

### Post-colonoscopy indicators

Concerning immediate complications in phase 1, there was no perforation and only one hemorrhage after polypectomy, which was treated during the same colonoscopy, without recurrence. In phase 2, there was one perforation in a context of diverticulosis without polypectomy, and no hemorrhage or inhalation. Information on delivery of the day-0 *mail* was not easily available for the 1st phase. We included the automatic generation of the letter in our electronic report. For outpatients, a day-*0 mail* and printed endoscopic report were generated for and delivered to only 24 patients (27%) during phase 1 versus 91 patients (100%) during phase 2 (*p* = 0.0001).

## Discussion

The main improvements in our practices were the development and standardization of our electronic colon preparation and examination report forms, and the fact that they were made available to all hospital practitioners on the intranet server. This new strategy led to an improvement in the quality of colon preparation for our patients and in the quality of our examination reports, with ESGE and SFED objectives largely achieved. These results are in accordance with previous interventional studies: standardization and implementation of a uniform bowel preparation procedure, and in particular split dosing, increased the proportion of “good” (more than 90% of mucosa visualized) and “excellent” (more than 95%) results from 84.6% to 86.5% with fewer “fair” results and no change for “poor” results (around 5%)^16^. In a cohort study of inpatients, the benefit of a colonoscopy preparation electronic order form favoring split dosing was shown, with 86% of good preparation versus 43% before the intervention^17^. Another study also showed the benefit of an electronic order form, as the proportion of colonoscopies with inadequate preparation was significantly reduced and the diagnostic yield modestly improved^18^. In addition, the use of an electronic order form increased the mention of BBPS scores in colonoscopy reports from 0.5 to 85%^17^ and favorably impacted compliance with follow-up guidelines in an average-risk screened population^19^. The implementation of an electronic colonoscopy preparation order set had the strongest impact in an interventional study^20^ that also included the education of physicians and patients. The overall result was a reduction in the rate of poor colonoscopy preparations from 19 to 4%.

The inclusion of a polyp description paragraph in the standardized endoscopy report probably explains the decrease in the proportion of hyperplastic polyp resections and the increased proportion of adenoma resections. The Paris classification is the main polyp classification used in our electronic endoscopic reports, even though the Kudo classification does not give the same information (crypt structure and so histology prediction). The preference can be explained by the simplicity of the Paris compared to the Kudo classification, which needs more time for precise examinations and electronic color enhancement. The Kudo classification was used only for large polyps and only by one practitioner, but should be used every time to predict polyp histology. In our study, practitioners provided predictions of polyp histology, but did not clearly use the Kudo classification in endoscopic reports.

ESGE and SFED recommend an ADR greater than 25% for diagnostic colonoscopies (IBD, planned polyp resections and emergencies excluded) and greater than 45% for FIT indications. In our study, even if this rate was not individual, this objective was reached in both phases, and also improved in phase 2. This increase in the ADR and PDR could probably be related to the improvement in the quality of colon preparation, in particular the use of split dosing, as shown by Seo et al.^21^. For FIT colonoscopy indications, the ADR cannot be considered because of the small number of colonoscopies performed, due to a temporary interruption of the mass screening program in France at the time of the study.

The withdrawal time item was included in the standardized colonoscopy report and was longer than 6 min (ESGE major criterion) in all cases.

Our new electronic colonoscopy report made it possible to generate a day-0 mail and a printed report, which was delivered to all outpatients when they go back home.

Time to the colonoscopy sadly increased between the two phases of our study. This could be explained by the development in our unit of advanced therapeutic colonoscopy techniques with more complex polyp resections. It is necessary to increase the number of endoscopy procedures under general anesthesia to reduce times to endoscopy access. The SFED strongly recommends a maximum of 31 days between an FIT gastroenterology visit and the colonoscopy^7,8^. We were close to this objective during the first phase of the study but we did not have enough patients with a positive FIT to show significant results. Proposals to reduce the time between specialist consultations and the colonoscopy would require dedicated consultation schedules and increased access to operating rooms (OR), but currently all OR slots are already saturated and the margin to increase their number is narrow.

The strengths of this study are the choice of internationally validated quality indicators with recent, clear recommendations. We also studied other non-validated but interesting indicators, such as times between the consultation with a gastroenterologist or anesthesiologist and the colonoscopy, to take stock of our practices and follow their evolution over time.

Nevertheless, there are several limitations in this work. First, the two populations were not exactly comparable in term of colonoscopy indications. This is simply explained by the smaller number of Fecal Immunologic Tests in France at the time of the second phase. This was a single-center study, our conclusions therefore cannot be generalized to all endoscopy centers. Each center has its own procedures, and these procedures must be assessed in each center to identify factors that could be improved before implementing specific procedures. Another limitation is the variability among physicians in rating the preparation. This could be a source of bias, even though we used the standardized, recognized BBPS. For the future, there is still room for progress, especially in colonoscopies with a BBPS score of 7 or more, and in cecal intubation rates, which should rise to 90–95%. Concerning adenoma detection rates, a greater number of patients are needed to make a robust analysis and obtain significant results. Withdrawal time is at least 6 min, but should tend towards 10 min, and the precise measurement of this time is needed.

## Conclusion

Our study allowed us to identify shortcomings in our procedures, and as a result to rapidly implement new corrective actions to increase the quality of our unit and our personal practices, particularly in the quality of colon cleansing, which led to an increase in the adenoma detection rate. In addition, our intervention resulted in an increase in the completion rates for several items, such as BBPS and the Paris classification, and the automatic generation of a day-0 *mail* and printed colonoscopy report. Our intervention required an initial analysis of the shortcomings of our unit and can therefore only be generalized to units that have identified the same shortcomings. However we think that this program is feasible and easy to implement, and results in the improved quality, safety and performance of a digestive endoscopy unit. Our study could serve as an example to encourage administrators of endoscopy units to undertake creative and effective actions to improve colonoscopy quality. This could easily be done in every digestive endoscopy unit.

Quality improvement is a steady, continuous effort, and we need to continue auditing and improving to get closer to what is best for our patients.

## Supplementary Information

Supplementary Information.
