# Supplementary material for: A colonoscopy quality improvement intervention in an endoscopy unit
Source: Sci Rep. 2022 Jan 17;12:817. doi: 10.1038/s41598-022-04786-y (PMC8763865; doi:10.1038/s41598-022-04786-y)
Supplement: Supplementary file 1 — Supplementary Information. [file 41598_2022_4786_MOESM1_ESM.docx]

Appendix : table of quality criteria studied

| Indicators | ESGE  objectives | SFED  objectives |
| --- | --- | --- |
| **Pre-procedure** |  |  |
| Specialist visit before colonoscopy | >90% | |
| Consent | 100% | |
| Endoscopic checklist including validation of endoscope disinfection | 100% | |
| Presence of a valid indication | >85%) | |
| Bowel cleansing regimen given | 100% | |
|  | Additional quality indicators | |
| Tolerance to bowel cleansing | Very good/Good/Medium/Bad/Very bad | |
| Time to colonoscopy |  | |
| **Per-procedure** |  |  |
| Boston Bowel Preparation Scale (BBPS) | >5  >90% | >6  >90% |
| Cecal intubation rate | >90% | |
| Withdrawal time measurement | ≥6 minutes | |
| Polyp description, Paris and Kudo classifications | Yes/No | |
| Polyp retrieval rate | >90% | |
| Adenoma detection rate (ADR) | ≥25%  ≥45% for FIT | |
| **Post-procedure** |  |  |
| Complications at day-0 |  | |
| Complete and detailed exam reports |  |  |
|  | Additional quality indicator | |
| Printed day-0 mail | Yes/No | |
